# Predictors of Major Adverse Cardiovascular and Cerebrovascular Events After Acute Coronary Syndromes: A Retrospective Observational Study Using YoMDB Database

**DOI:** 10.14789/jmj.JMJ22-0049-OA

**Published:** 2023-07-24

**Authors:** YOSHINORI HAMA, HIROAKI ITOH, SACHIKO NAKAGAMI, TAIGA CHIBA, SOSHI DOHMAE, YUJI NISHIZAKI, SHUKO NOJIRI, YUKIO SUZUKI, KAZUHITO YOKOYAMA

**Affiliations:** 1 Department of Epidemiology and Environmental Health, Juntendo University Graduate School of Medicine, Tokyo, Japan; 1 Department of Epidemiology and Environmental Health, Juntendo University Graduate School of Medicine, Tokyo, Japan; 2 Department of Epidemiology and Environmental Health, Juntendo University Faculty of Medicine, Tokyo, Japan; 2 Department of Epidemiology and Environmental Health, Juntendo University Faculty of Medicine, Tokyo, Japan; 3 Medical Policy Division, Medical Care Bureau, Kanagawa, Japan; 3 Medical Policy Division, Medical Care Bureau, Kanagawa, Japan; 4 Division of Medical Education, Juntendo University School of Medicine, Tokyo, Japan; 4 Division of Medical Education, Juntendo University School of Medicine, Tokyo, Japan; 5 Medical Technology Innovation Center, Juntendo University, Tokyo, Japan; 5 Medical Technology Innovation Center, Juntendo University, Tokyo, Japan; 6 Clinical Research and Trial Center, Juntendo University, Tokyo, Japan; 6 Clinical Research and Trial Center, Juntendo University, Tokyo, Japan; 7 Department of Obstetrics and Gynecology, Yokohama City University Graduate School of Medicine, Kanagawa, Japan; 7 Department of Obstetrics and Gynecology, Yokohama City University Graduate School of Medicine, Kanagawa, Japan; 8 Division of Gynecologic Oncology, Department of Obstetrics and Gynecology, Columbia University Vagelos College of Physicians and Surgeons, New York, USA; 8 Division of Gynecologic Oncology, Department of Obstetrics and Gynecology, Columbia University Vagelos College of Physicians and Surgeons, New York, USA; 9 Department of Epidemiology and Social Medicine, International University of Health and Welfare Graduate School of Public Health, Tokyo, Japan; 9 Department of Epidemiology and Social Medicine, International University of Health and Welfare Graduate School of Public Health, Tokyo, Japan

**Keywords:** stroke, myocardial infarction, care, nursing

## Abstract

**Objectives:**

Despite the rapid aging of the population in Japan, clinical predictors for major adverse cerebrovascular and cardiovascular events (MACCE) in patients with new onset of acute coronary syndromes (ACS) have not been well studied. This study therefore aimed to identify the predictors of MACCE in the first onset of ACS patients requiring care.

**Materials and Methods:**

Using the Yokohama Original Medical Database, we identified 3,373 patients who experienced a first onset of ACS and had certified care information from April 2014 to March 2016. The incidence proportion of MACCE from June 2014 to March 2018 was retrospectively investigated. Each patient's independence of daily living (IDL) was classified as one of three categories (reference, mild and severe).

**Results:**

Predictors of MACCE were identified using multivariate logistic regression analysis. Impaired IDL was associated with increased MACCE, with adjusted odds ratios for reference, mild and severe of 1.00, 1.35 (95% confidence intervals 1.14-1.60) and 2.12 (95% confidence intervals 1.61-2.80; P for trend < 0.001), respectively.

**Conclusions:**

This study revealed that male sex, chronic kidney disease, atrial fibrillation, high-intensity statin use, low-intensity statin use, and lower IDL (representing less independence) were the predictors of MACCE requiring care for a first onset of ACS. Further research will be required to understand the results of interventions for the identified predictors of MACCE.

## Introduction

The role of long-term care insurance has become more important in Japan with the emergence of its super-aging society. The number of persons certified as requiring care under the long-term care insurance system has increased approximately 2.5 times since the start of the long-term care insurance system in 2000, from 2.56 million persons to approximately 6.41 million persons in 2018, and long-term care benefit expenditure has increased approximately 2.9 times, from 3.2 trillion yen ($23.7 billion) to 9.2 trillion yen ($68.1 billion), showing an especially rapid increase in the most recent data^1)^. The average life expectancy of Japanese people is among the highest in the world. However, healthy life expectancy is considerably shorter, with a gap of approximately 9 years for men and approximately 12 years for women, meaning that many elderly people require support and care for some 10 years in late life. Cerebral and cardiovascular disease account for 16.6% and 4.6%, respectively, of the causes leading to this need for care, together representing the highest proportion, 21.2%, from any reported cause^2)^. Therefore, the prevention of cerebral and cardiovascular disease is important for the suppression of expenditures on national healthcare and long-term care benefits.

National healthcare expenditure in Japan continues its increasing trend, and total national healthcare expenditure in 2018 was 43 trillion yen ($319 billion)^2)^. When medical expenses are examined by injury/disease category resulting from primary injury/disease, cerebral and cardiovascular disease is the most expensive category, at 6.1 trillion yen ($44.9 billion; composition ratio: 19.3%), and among both men and women represents the greatest impact on medical expenses.

Among cardiovascular diseases, ischemic heart disease in the form of acute coronary syndromes (ACS) is often the cause of heart failure. Several studies conducted in Japan in patients with acute heart failure have reported that ischemic heart disease accounts for more than 30% of the major causative diseases leading to acute heart failure^3-7)^. In another report of 7,733 patients aged 65 years or older who were hospitalized for a first onset of myocardial infarction without a history of heart failure, approximately 75% of patients developed heart failure within 5 years, and approximately 40% of them died^8)^. These reports suggest that prevention of ACS may also contribute to preventing the development of heart failure.

In a recent registry study of ACS in Japan, 27%-40% of patients with ACS were reported to have cerebral or cardiovascular events within 3 years^9)^. Independent predictors for 3-year major adverse cardiac events have been reported as higher age, dyslipidemia, chronic kidney disease, stroke, peripheral artery disease, previous myocardial infarction, and Killip class ≥ 2^10)^. In addition, a prospective multicenter study investigating the prevalence and clinical outcomes of polyvascular disease including stroke in patients with ACS showed that ACS patients with polyvascular disease had poorer clinical outcomes, including recurrent myocardial ischemia, heart failure, stroke, and death, than did those without polyvascular disease^11)^. Given these reports, suppression of recurrence of ACS or stroke means improving patient outcomes, but additionally has broad and important socio-economic implications. This concept is also consistent with addressing “secondary prevention” as outlined in the Cerebrovascular and Cardiovascular Disease Control Act, enacted in Japan in December 2018 as the first ever legislative countermeasure against stroke and cardiovascular disease in Japan, and is an important consideration for effective evidence-based policy making^12)^.

However, to our knowledge there has been no report on the associations between the recurrence of cerebral and cardiovascular disease and the degree of independence in daily living (IDL). Yokohama, one of the largest cities in Japan, has its own medical database, Yokohama Original Medical Database (YoMDB), based on long-term care insurance. Therefore, the present study was conducted using this database to clarify the clinical setting of major adverse cerebral and cardiovascular events (MACCE) and the predictors for MACCE in patients undergoing care for the first onset of ACS.

## Materials and Methods

### Database

Medical claims and long-term care data since April 2014 from YoMDB were used for this study^13)^. YoMDB is a large medical invoice database that includes residents of Yokohama City who have one of the following three insurance types: National Health Insurance, Medical Care System for Older Senior Treatment, and Public Assistance. Data for a single year include more than 30 million records, covering 86.4% of the entire population aged 65 years or older and more than 99% of the population aged 75 years or older in Yokohama City, where nearly 3.7 million people live. The total data for 2015 included 1,247,408 people and 14,944,496 medical invoices. Based on YoMDB's use policy, values less than 10 and those that were less than 10 after subtraction were omitted to avoid potential identification of individuals. This study used an opt-out system at the official website of Yokohama City, instead of obtaining informed consent from patients.

### Subjects

The subjects included patients who received certification of need for support or long-term care at the end of each fiscal year from June 2014 to March 2018 and who had new onset of ACS during the 2 years from April 2014 to March 2016. Outcomes were MACCE between June 2014 and March 2018. MACCE was defined as all-cause death, ACS, or stroke with hospitalization receipt data for the primary disease name within 2 years after the onset of ACS. Patients with MACCE within 30 days after the first onset of ACS were excluded, given that there may be patients who have been hospitalized since the previous month. A first onset of ACS was defined as occurring in patients who had no ACS prior to 2014 and had ACS initiated between April 2014 and March 2016. For the purposes of patient background characteristics, the baseline was defined as the time of the first onset of ACS.

**Figure 1:**
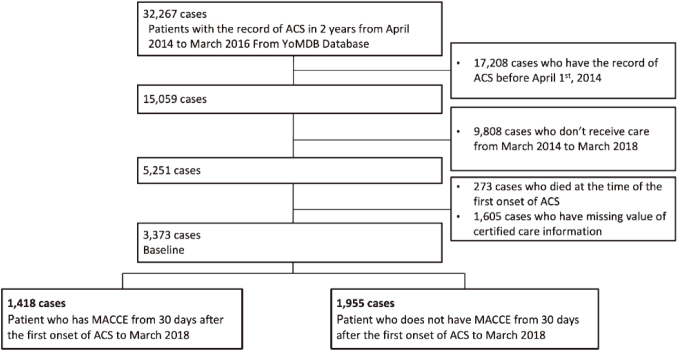
Study flow chart ACS indicates acute coronary syndromes; YoMDB, Yokohama Original Medical Database; and MACCE, major adverse cerebrovascular and cardiovascular events. A total of 1,605 cases were excluded due to lack of certified care information, i.e., IDL rank.

The study flow chart and baseline characteristics are shown in Figure 1 and Table 1, respectively. From a total of 32,267 patients in the YoMDB database who had a record of ACS in the 2 years from April 2014 to March 2016, after excluding 17,208 cases who had ACS recorded before April 1, 2014, we identified 15,059 cases. We then excluded 9,808 cases who did not receive care from March 2014 to March 2018; 5,251 cases remained. From these were excluded 273 subsequent cases that occurred within one month after the first onset of ACS and 1,605 patients for whom care certification information was not obtained throughout the observation period. Finally, 3,373 cases were extracted as the analysis set.

**Table 1:** Baseline characteristics of 3373 subjects

Baseline characteristics (At first onset of ACS)	n	%
Age, mean (±SD) [years]	82.8 (± 7.4)	
≤ 64 years	56	1.7%
65-74 years	344	10.2%
75-84 years	1,510	44.8%
≥ 85 years	1,463	43.4%
Male sex	1,459	43.3%
Comorbidity (n, %)		
Stroke		
Stroke all	1,010	29.9%
Ischemic stroke	977	29.0%
Hemorrhagic stroke	55	1.6%
Peripheral artery disease (PAD)	109	3.2%
Hypertension	2,924	86.7%
Diabetes	1,040	30.8%
Dyslipidemia	1,603	47.5%
Carotid artery stenosis	290	8.6%
Chronic kidney disease (CKD)	421	12.5%
Atrial fibrillation (AF)	886	26.3%
Aortic valve stenosis	114	3.4%
Dementia	861	25.5%
Surgery (n, %)		
Coronary revascularization		
Total	418	12.4%
PCI (Percutaneous coronary intervention)	405	12.0%
CABG (Coronary artery bypass grafting)	13	0.4%
Transportation by ambulance (n, %)	1,101	32.6%
Medication (n, %)		
ARB (angiotensin II receptor blocker)	1,015	30.1%
ACEi (angiotensin converting enzyme inhibitor)	228	6.8%
Beta-blocker	496	14.7%
Low intensity statin	465	13.8%
High intensity statin	764	22.7%
Dementia drugs	407	12.1%
Antiplatelet/antithrombotic	2,301	68.2%
Single	1,389	41.2%
Double	826	24.5%
Triple	86	2.5%
Independence of daily living		
Category (Rank) (n, %)		
Reference (Independent, I, IIa)	2,080	61.7%
Mild (IIb, IIIa, IIIb)	1,015	30.1%
Severe (IV, M)	266	7.9%
Unknown	12	0.3%

### Data extraction: Classification based on the IDL

The degree of IDL was based on the attending physician's opinion on “Degree of IDL for the elderly with dementia” recorded in the long-term care insurance certification information and was classified into one of three groups (Supplemental Table 1). Patients who were able to independently take medications were categorized as the Reference group, those needing assistance in taking medications as the Mild group, and the severely disabled as the Severe group. This status of independence was used as an explanatory variable in a multivariate logistic regression analysis.

### Data extraction: Medical history, comorbidities, procedure, and IDL

Past medical history and comorbidities were extracted when there was a claim code indicating medical history and comorbidities prior to the first claim of ACS. Surgical procedure data were extracted when it was claimed in the same month as the first onset of ACS. Medications were extracted if they were prescribed in the month when the index disease started. The degree of IDL was extracted at the time point closest in end of March and September to the time of first onset of ACS.

### Data extraction: Medication

Medication status was extracted when there was any prescription filled at discharge or pharmacy receipt on record. Details of definition and classification of each medication are shown in Supplemental Table 2.

### Data extraction: ICD-10 code

The ICD-10 code for each disease name and procedure was extracted with details shown in Supplemental Table 3.

### Statistical analysis

Predictors, odds ratios, and 95% confidence intervals of MACCE were analyzed using logistic regression analysis according to baseline characteristics. All independent variables, selected in accordance with the related research to date, were simultaneously introduced, and the incidence proportion of MACCE was used as a dependent variable. Missing values were handled by complete case analysis. P values of <0.05 were considered statistically significant. EZR statistical software (version 4.0.3), which extends the functionality of R and R Commander software, was used for all statistical analyses^14)^.

### Ethical considerations

This study was conducted with the approval of the Juntendo University Institutional Review Board (Approval No. M20-0256-M01).

## Results

### Incidence proportion of MACCE

**Figure 2:**
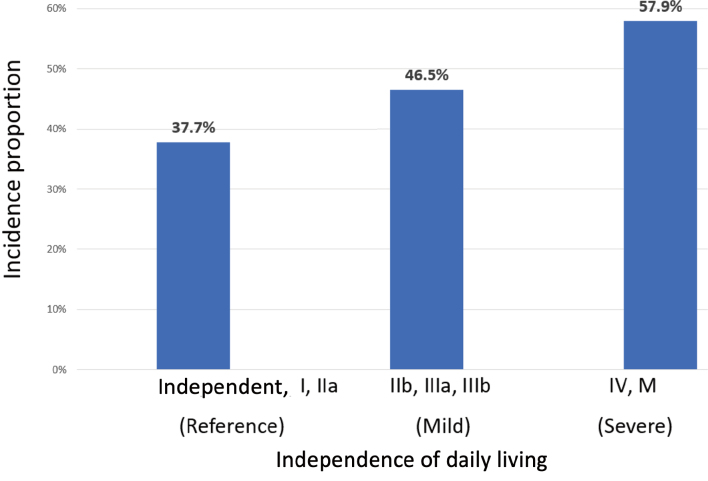
Rank of IDL and incidence proportion of MACCE IDL rank and incidence proportion of MACCE. This analysis excludes 12 patients whose degree of independence of daily life was unknown. Classification of IDL rank is shown in Supplemental Table 1. IDL, independence of daily living; MACCE, major adverse cerebrovascular and cardiovascular events.

The incidence proportion of MACCE and baseline characteristics according to presence or absence of MACCE after follow-up are shown in Table 2. The incidence proportion of MACCE was 42.0% in patients with the first onset of ACS. Patients were classified into the 1,418 patients who had MACCE after 30 days from the first onset of ACS until March 2018, and the 1,955 patients who did not have MACCE during the above period. The incidence proportion of MACCE according to IDL rank are shown in Figure 2. The incidence proportion of MACCE in reference, mild and severe were 37.7%, 46.5% and 57.9%, respectively (P for trend < 0.001 by Cochran-Armitage test).

**Table 2:** Incidence proportion of MACCE and baseline characteristics according to presence or absence of MACCE during follow-up

	MACCE			
	Presence	%	Absence	%
All cases (n=3,373) (n, %)	1,418	42.0%	1,955	58.0%
Age (mean ± SD) [years]	83.6 ± 7.8		82.3 ± 7.1	
Age (n, %)				
≤ 64 years	23	1.6%	33	1.7%
65-74 years	135	9.5%	209	10.7%
75-84 years	580	40.9%	930	47.6%
≥ 85 years	680	48.0%	783	40.1%
Male (n, %)	718	50.6%	741	37.9%
Comorbidity (n, %)				
Stroke				
Stroke all	493	34.8%	517	26.4%
Ischemic stroke	475	33.5%	502	25.7%
Hemorrhagic stroke	30	2.1%	25	1.3%
Peripheral artery disease (PAD)	46	3.2%	63	3.2%
Hypertension	1,223	86.2%	1,701	87.0%
Diabetes	437	30.8%	603	30.8%
Dyslipidemia	618	43.6%	985	50.4%
Carotid artery stenosis	126	8.9%	164	8.4%
Chronic kidney disease (CKD)	229	16.1%	192	9.8%
Atrial fibrillation (AF)	432	30.5%	454	23.2%
Aortic valve stenosis	52	3.7%	62	3.2%
Dementia*	396	27.9%	465	23.8%
Surgery (n, %)				
Coronary revascularization				
Total	152	10.7%	266	13.6%
PCI (Percutaneous coronary intervention)	147	10.4%	258	13.2%
Transportation by ambulance (n, %)	486	34.3%	615	31.5%
Medication (n, %)				
ARB (Angiotensin II receptor blocker)	410	28.9%	605	30.9%
ACEi (Angiotensin converting enzyme inhibitor)	92	6.5%	136	7.0%
Beta-blocker	186	13.1%	310	15.9%
Low intensity statin	142	10.0%	323	16.5%
High intensity statin	284	20.0%	480	24.6%
Dementia drugs	179	12.6%	228	11.7%
Antiplatelet/antithrombotic	990	69.8%	1,311	67.1%
Single	606	42.7%	783	40.1%
Double	352	24.8%	474	24.2%
Triple	32	2.3%	54	2.8%
Independence of daily living				
Category (Rank) (n, %)				
Reference (Independent, I, IIa)	785	55.4%	1,295	66.2%
Mild (IIb, IIIa, IIIb)	472	33.3%	543	27.8%
Severe (IV, M)	154	10.9%	112	5.7%

*: Dementia, as comorbidity, was derived from claim database, which was diagnosed by clinical physician.

### Multivariate analysis

**Figure 3:**
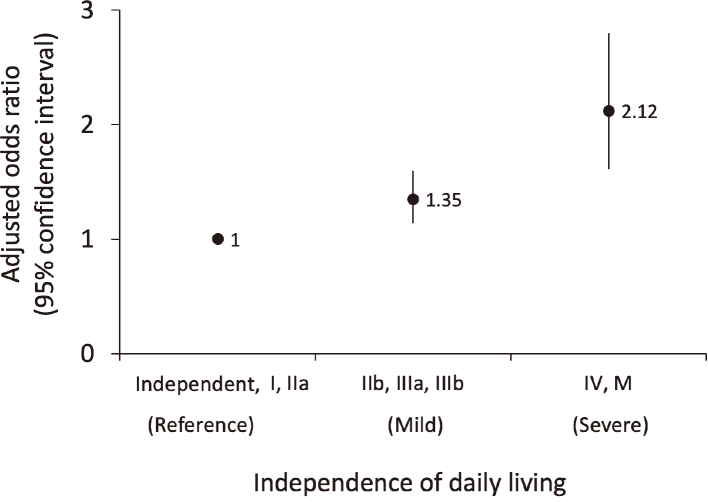
IDL category and adjusted odds ratios of MACCE IDL category and adjusted odds ratios of MACCE with 95% confidence interval. This analysis excludes 12 patients whose degree of independence of daily life was unknown. Classification of IDL category is shown in Supplemental Table 1. IDL, independence of daily living; MACCE, major adverse cerebrovascular and cardiovascular events.

The multivariate logistic regression analysis showed that male sex, concurrent chronic kidney disease, concurrent atrial fibrillation, taking high- intensity statins, taking low-intensity statins, and severe IDL (less independence) are independent predictors of MACCE (Table 3). Positive associations were observed between male sex, concurrent chronic kidney disease, concurrent atrial fibrillation, and MACCE. A positive association was also observed between the category of IDL and MACCE. Contrast negative associations were observed between taking high-intensity statins, taking low-intensity statins, and MACCE. Adjusted odds ratios and 95% confidence intervals (CI) for reference, mild and severe IDL were 1.00, 1.35 (95% CI 1.14-1.60) and 2.12 (95% CI 1.61-2.80; P for trend < 0.001), respectively (Figure 3).

**Table 3:** Odds ratios and 95% confidence intervals (CIs) of MACCE according to baseline characteristics by multivariate logistic regression analysis

Independent variables	Univariate				Multivariate			
	Odds ratio	95% CI lower limit	95% CI upper limit	p-value	Odds ratio	95% CI lower limit	95% CI upper limit	p-value
Gender								
Female	Referent				Referent			
Male	1.68	1.46	1.93	<0.001	1.66	1.43	1.92	<0.001
Age								
≤ 64 years	Referent				Referent			
65 -74 years	0.92	0.52	1.63	0.76	1.14	0.63	2.06	0.67
75 -84 years	0.89	0.52	1.54	0.68	1.22	0.69	2.15	0.48
≥ 85 years	1.25	0.72	2.14	0.42	1.69	0.95	2.98	0.072
Comorbidity*								
Hypertension	0.94	0.77	1.15	0.52	0.96	0.77	1.19	0.68
Diabetes	1.00	0.86	1.15	0.94	1.04	0.88	1.22	0.64
Dyslipidemia	0.76	0.66	0.87	<0.001	0.88	0.75	1.03	0.10
Peripheral artery disease (PAD)	1.03	0.70	1.51	0.89	1.11	0.74	1.66	0.62
History of stroke all	1.49	1.29	1.73	<0.001	1.84	0.89	3.78	0.098
History of ischemic stroke	1.47	1.26	1.70	<0.001	0.79	0.38	1.63	0.51
Carotid artery stenosis	1.07	0.84	1.37	0.56	1.00	0.77	1.30	0.98
Chronic kidney disease (CKD)	1.76	1.43	2.16	<0.001	1.76	1.41	2.18	<0.001
Atrial fibrillation (AF)	1.44	1.24	1.68	<0.001	1.31	1.11	1.55	0.001
Aortic valve stenosis	1.17	0.80	1.70	0.42	1.30	0.88	1.93	0.18
Dementia	1.24	1.06	1.45	0.006	1.04	0.84	1.29	0.71
Transportation by ambulance	1.14	0.98	1.32	0.086	1.05	0.90	1.23	0.53
Medication*								
ARB (Angiotensin II receptor blocker)	0.91	0.78	1.06	0.21	0.98	0.84	1.16	0.83
ACEi (Angiotensin converting enzyme inhibitor)	0.94	0.71	1.23	0.64	0.99	0.74	1.32	0.93
Beta-blocker	0.80	0.66	0.98	0.028	0.86	0.70	1.06	0.15
Low-intensity statin	0.56	0.46	0.69	<0.001	0.59	0.47	0.75	<0.001
High-intensity statin	0.77	0.65	0.91	0.002	0.80	0.66	0.98	0.028
Dementia drugs	1.10	0.89	1.36	0.36	0.93	0.71	1.22	0.60
Antiplatelet/antithrombotic Single	1.11	0.97	1.28	0.12	1.08	0.91	1.30	0.37
Antiplatelet/antithrombotic double	1.03	0.88	1.21	0.68	1.18	0.95	1.47	0.13
Antiplatelet/antithrombotic Triple	1.17	0.80	1.70	0.42	1.30	0.88	1.93	0.18
IDL: Independence of daily living								
Category: **Reference** (Rank: Independent, I, IIa)	Referent				Referent			
Category: **Mild** (Rank: IIb, IIIa, IIIb)	1.43	1.23	1.67	<0.001	1.35	1.14	1.60	<0.001
Category: **Severe** (Rank: IV, M)	2.27	1.75	2.94	<0.001	2.12	1.61	2.80	<0.001

Objective variable: MACCE *Referent of variables in comorbidity and medication are no comorbidity and no medication.

## Discussion

In the present study, risk factors for MACCE were determined using highly complete real-world data from YoMDB database. A multivariate logistic regression analysis showed that male sex, concurrent chronic kidney disease, concurrent atrial fibrillation, high-intensity statin use, low-intensity statin use, and a high category of IDL (signifying less independence) were predictors of MACCE. Also, degree of IDL was associated with the incidence proportion of MACCE.

### Predictors of MACCE

The predictors of MACCE revealed in this study are in accord with previous research. Many studies have shown that the incidence proportions of MACCE and their sequelae are higher in males than in females. Similarly, CKD is a known high-risk condition of arteriosclerotic disease that has been associated with an increased risk of cardiovascular events^15, 16)^. Atrial fibrillation is associated with increased cardiovascular risk^17)^, and many previous studies have reported the effect of statins on MACCE^18)^. We may need to consider the background and severity of patients when interpreting the odds ratios in the high-intensity statin group and the low-intensity statin group in this study, as the odds ratios seem to be the inverse of what would be anticipated, i.e., this may be because severe patients in whom the risk of MACCE is difficult to reduce are prone to use high-intensity statin because of its complexity. These previously known risk factors were confirmed as predictors of MACCE in this population with newly diagnosed ACS requiring interventional care.

### Relationship between long-term care and MACCE

According to National Health Care Coverage data, in fiscal year 2018, the total percentage of causative diseases requiring support/long-term care was 18.5% for cerebrovascular disease and 4.5% for cardiovascular disease, and the total number for cerebral and cardiovascular diseases was 23.0%. The proportion increases as the level of need for long-term care rises. Cerebral and cardiovascular disease accounted for the highest proportion, 35.9%, of the level 5 cases requiring long-term care, which is the highest care requirement^19)^. Furthermore, it has been reported that the level of long-term care required often progresses irreversibly in a stepwise manner from support to long-term care needs^20)^.

Given these reports, the secondary occurrence of cerebral and cardiovascular events is one of the factors influencing the progression of the level of care needed. Therefore, it may be important to prevent recurrence or subsequence of cerebral and cardiovascular events to prevent this progression.

### Relationship between IDL and MACCE

Prior reports on patient frailty may be of use in interpreting the relationship between the degree of independence of daily living and cerebral and cardiovascular events revealed in this study. Frailty, recently identified as being characteristic of the physical and mental state of the elderly, is defined as a state intermediate between health and requiring care^21)^. Frailty has been shown to be associated with impaired activities of daily living, hospitalization, and life expectancy, and the presence of frailty has also been reported as a risk factor for certification of long-term care needs^22)^. Patients with cardiovascular disease have a 2.7-4.1-fold higher prevalence of frailty and a 1.5-fold higher longitudinal incidence; in addition, patients with coronary artery disease or heart failure who also have frailty have a 1.6-4-fold increased risk of death^23)^.

Thus, the relationship between frailty and cerebral and cardiovascular diseases has already been clarified in several studies. In other words, the condition in which daily life is more independent may lead to the suppression of subsequent cerebral and cardiovascular events, and interventions for the controllable predictors shown in this study may aid in the suppression of progression to needing a greater level of care. Furthermore, such interventions for suppression of MACCE may also extend healthy life expectancy.

### Limitations of this study

This study has several limitations. First, there is a possibility of incurring some selection bias by limiting cases to those for which long-term care certification information is available. Second, detailed information such as smoking habits, body mass index, and various laboratory test values are unavailable in the YoMDB database, and the severity and control status of ACS and each comorbidity are not taken into consideration. Third, each comorbidity and medication selected as an explanatory variable may have been over-evaluated because of the criteria to pick up, i.e., the existence of any prescription filled, and no validation for that. Forth, this study has no detailed information on ADL such as Barthel Index, which is crucial to fully understand the association between MACCE and frailty. Fifth, the association between MACCE and IDL as history of included stroke patients may have some bias, even though this was adjusted as an explanatory variable in the logistic regression analysis. Finally, further research will be required to understand the results of interventions for the identified predictors of MACCE, as investigating the clinical impact of such interventions is beyond the scope of this study.

## Conclusion

In patients with a first onset of ACS, male gender, chronic kidney disease, atrial fibrillation, high-intensity statin use, low-intensity statin use, and lower IDL were predictors of MACCE. The higher level of care a patient needed, the more likely MACCE was to occur. Therefore, given the current and projected future care needs, as well as previous studies, interventions for controllable factors may be effective in reducing the incidence of MACCE in patients with the first onset of ACS.

## Funding

The authors received no financial support for the research.

## Author contributions

YH was a major contributor in writing the manuscript. SN (Sachiko Nakagami), TC, and SD provided YoMDB database and performed statistical analysis. HI, YN, SN (Shuko Nojiri), and YS provided advice and guidance. KY supervised this study and provided advice and guidance. All authors read and approved the final manuscript.

## Conflicts of interest statement

YH is an employee of Amgen K.K.

**Supplemental Table 1:** Classification based on the independence of daily living (IDL)*

Code	IDL rank	Category
1	Independent	1: Reference
2	I	
3	IIa	
4	IIb	2: Mild
5	IIIa	
6	IIIb	
7	IV	3: Severe
8	M	

*: “Degree of IDL for the elderly with dementia” was referred for this study because it was more suitable for the population of this study than “Degree of IDL for the disabled elderly”.

**Supplemental Table 2:** Definition and classification of each medication

Classification	Definition
High-intensity statin	Any of daily atorvastatin ≥ 10 mg, pitavastatin ≥ 2 mg, rosuvastatin ≥ 5 mg, simvastatin ≥ 20 mg, fluvastatin ≥ 80 mg, pravastatin ≥ 40 mg, or any statin (even at low intensity) plus ezetimibe ^24)^.
Antithrombotic drugs: Single therapy	An oral anticoagulant (OAC) alone (warfarin, dabigatran, rivaroxaban, apixaban, or edoxaban) or single antiplatelet therapy (SAPT)(aspirin/P2Y12 receptor blocker (ticlopidine, clopidogrel, prasugrel, or ticagrelor)) ^25)^.
Antithrombotic drugs: Double therapy	OAC plus clopidogrel/prasugrel or dual antiplatelet therapy (DAPT) ^25)^.
Antithrombotic drugs: Triple therapy	OAC plus DAPT. Two or more OACs were regarded in the same manner as one drug, and SAPTs other than clopidogrel/prasugrel were regarded as clopidogrel/prasugrel ^25)^.

**Supplemental Table 3:** ICD-10 code of each disease name and procedure

Disease	ICD-10 code
Acute coronary syndrome	I 20.0, I 21.1-21.4, I 21.9, I 22.0, I 22.1, I 22.8-9, I 24.9
Heart failure	I 50.0-50.1, I 50.9, I 11.0, I 13.0, I 13.2
Pulmonary embolism	I 26.0, I 26.9
Acute aortic dissection or aortic aneurysm	I 71.0-71.9
Diabetes mellitus (type 1 and 2 diabetes mellitus)	E 10-11
Atrial fibrillation	I 480-482, I 489
Dementia	G 300-301, G 308-309, G 318, F 019, F 03
Alzheimer dementia	G 300-301, G 308-309
Dementia with Lewy Bodies	G 318
Cerebrovascular dementia	F019
Hypertension	I 10
Peripheral artery disease	I 739
Stroke	I 619, I 639, I 64
Chronic kidney disease	N 189
Dyslipidemia	E 785
Pneumonia	J 10-18
Malignant tumor	C 00-C 97
Cerebral hemorrhage	I 619
Cerebral infarction	I 638-639, I 64
PCI	K 546-550, K 550-2
CABG	K 552, K 552-2
